# Food, Health and Preventive Dentistry*Abstract of an address delivered before the National Dental Association and printed in the April Cosmos.

**Published:** 1913-05-15

**Authors:** Harry W. Wiley


### FOOD, HEALTH AND PREVENTIVE DENTISTRY.*

### FOOD AS OF PRIME IMPORTANCE TO HUMANITY.

BY HARRY W. WILEY, M.D.

Eating is the greatest industry of humanity; we know that it is widely pursued by all civilized nations, and those of the missionaries who come back tell us that it is practiced also by the savage cannibal. The principal outlet, I believe, for the salaries of most laboring men and of all wage earners of every description is to supply the wants of their bodies and their families' bodies; therefore I say it is the principal

---

*Abstract of an address delivered before the National Dental Association and printed in the April Cosmos.

industry of humanity, and it is that industry which requires of man the greatest amount of his effort.

Granting for the moment that this is a true statement, it follows that everything connected with that industry is of prime importance, and I take it that all agree with me that, after the cook, the teeth serve the most important function in the nutrition of man—even more so than the cook, for if we have good teeth we can eat raw foods and do not need a cook.

I am not a believer in that theory according to which one can chew a very little bit of food long enough to make up for a square meal. I believe in good chewing, and a lot of it. At dinner a very good friend of mine—a man who is somewhat of a valetudinarian and who devotes a great deal of time to thinking what he shall eat for his best welfare—and myself were discussing the subject of nutrition, and he said that the least lapse from the regular course of living might be followed by serious incapacity. I said that I was sorry he had to be so careful; that I for one gave no thought to what I was to eat; that I had followed the scriptural advice about the morrow, as to "what ye shall eat and wherewithal ye shall be clothed"—I let other people worry about that. "Sufficient unto the day," it is further said, "is the evil thereof"—and sufficient unto the day is the food thereof; the thing is, to get enough of it.

We hear a great deal at the present time about medical inspection in public schools, and no one believes in this more than I do. Whatever we may think ourselves about the matter, the fact remains that we are becoming every year more and more socialistic, becoming confirmed more and more in the belief that the state owes us something as citizens; everybody is becoming more sensitive to the idea that the state must care more than it has been doing heretofore for its citizens. We realize it today in the public schools. What right has the state to establish public schools under the doctrine of human rights? None, whatever. The citizen must care for himself, and the state has no right to interfere with his education; and yet what would we think of the person who would preach today the doctrine that public education was wrong? No, it is right, and public education is but a step toward socialism—the state does for the citizen what the citizen may do for himself, according to the theory of human rights. We are adopting that idea in education, as well as in the matter of public roads, so that we can all drive automobiles—at least those who have been in practice a few years can do it; our poor farmers of the East cannot do it yet, but they do out West—even if they have to put a mortgage on the farm. Thus we are more and more adopting the doctrine that the state must care for our opportunities. We are also coming to realize that the state must care for the health of the public, and we are coming to it rapidly.

When are we going to begin to care for the health? I should say that in the first years of toothhood—this is a new name!—we should begin to care for the health. The baby's mouth should be as carefully cleansed as the adult's every time it takes a square meal. The little tooth that just puts forth its appearance—and I have seen two in the last month that are the sweetest little teeth you ever saw—must be cared for. I can assure you that I never have taken so deep an interest in dentistry as in the last few months. I have not only made one examination, but about a hundred, to try to see if these young teeth were all right. So I say, in the first days of toothhood the care of the public health should begin. The state should see to it that its citizens are taught these principles of oral hygiene, which they can apply to their own infants in the beginning of their toothhood years. You may say that these teeth are only deciduous. That is true, but there is no reason why they should be lost by decay, because decay is contagious, and if it attacks the deciduous teeth there may be an infection left for the permanent ones, and then those permanent ones may be only as "temporary" as the first. And yet how careless we are, every one, of these first teeth.

Last year, while visiting my farmer, who has three little girls ranging from three to eleven years of age, I noticed that they paid no attention to their teeth at all, and so, in the goodness of my heart, I gave them tooth-brushes and told them how to use them. Some time after that I was back at the farm again. and I asked one of the little girls if she used her tooth-brush three times a day as suggested. "Oh, no," was her reply, "we are going to save them until we become grown girls"—then, of course, there would not be anything to brush. That was her idea of the way to use the gift; she would not use it in her childhood days, but would wait until she was grown and had no teeth. That is the way people regard the ordinary fundamentals of public health. The important thought to impress on them is that we cannot have good health without good teeth, and yet how often we leave the teeth to decay and be thrown on the scrap-heap early in life! I have seen in the year past girls of fifteen and sixteen years with teeth so badly decayed that they were almost as good as gone, simply owing to negligence on the part of their parents. When I was a child nothing was heard in the public schools of the idea of taking care of the teeth. It was not mentioned in the public schools of that locality. We were taught hygiene of the body in a way, some primitive ideas of living, but never a word of that most important part of personal hygiene, viz, starting infant as well as adult life with a good set of teeth.

Not everyone is born, like Richard Coeur de Lion, with a full set of teeth. That only happens to geniuses, but of course the brighter they are, the sooner they have teeth. My son has two teeth at four months of age. But whether they erupt soon or late, that is the time to begin to brush them. The teeth are like other blessings. We all know how "blessings brighten as they take flight," and I never appreciate a tooth so much as when I see it coming out of my mouth in the hands of a dentist, because I realize that it is through my negligence that I have lost it. There is no physiological reason why a man should ever loose his teeth; there is no reason why he should not keep them until old age, and I can assure you that, to my mind, there is no better way of reaching old age than by the preservation of the teeth.

For this reason, while I approve of medical inspection in schools, I approve also of dental inspection. Although this is no new idea, I insist that we should care for the deciduous teeth, and preserve them before the child goes to school. The state can hardly go into the homes and command that we shall preserve our children's teeth, but we can preach at least to the rising generation so that when they become parents they will realize the importance of tooth conservation. We cannot perhaps save the teeth that are erupting now, but we can do so twenty or forty years from now, just I as preach the doctrine of saving infant's lives. We cannot save the infants dying at the rate of a thousand a day now, but we can save those that will be borne forty years from now by beginning to preach infant hygiene today, so that the new generation will grow up with a new idea and a new purpose, namely to preserve intact as long as possible that precious endowment that has come to us, which we call life—that mystery which we cannot fathom, that heavenly gift which we should appreciate so much as to care for it so that we may get the most possible out of it. Therefore what I say now is not for the children of today—it is too late for them, but for the next ten, twenty and thirty years, when the child will be taught at the family fireside the principles of personal hygiene before he goes to school. As I have been preaching for the last third of a century the health of the public and the importance of preserving human life chiefly by means of proper nutrition, so I have more and more become convinced of the extreme necessity of saving the teeth for the purpose for which nature intended them.

This brings me to the fact that our country is being flooded with preparations which are prechewed and predigested; all kinds of infant, invalid, and breakfast foods, the manufacturers of which have taken the trouble to perform the functions not only of mastication but also of digestion. By this feeding of mankind with partially digested food, what happens? Just what happens to everything that is not in use. The next time you take a trip on a railroad, look out at the silver-like appearance of the rail that is in use, and then the one at the siding which is rusting away. Both tracks are made of the same steel, probably put down at the same time and in the same way, but one is bright and shining, and the other is rusting away because it is not in use. So it is with every organ of the human body; there is a legitimate physiological function for it to perform, and the law of all nature is, that if an organ fails to perform the function that nature intended it to perform, its function diminishes in efficiency, and every time we take prechewed and predigested food we injure the teeth and the stomach. After I have lost my teeth I will thank the manufacturer for chewing for me—until I can raise money enough to get an artificial set. After my stomach is gone and is no longer able to perform its functions, then I will thank him for digesting my food for me; but as long as I am in my present condition—I still have a few teeth left, and also a stomach which produces every day a certain amount of pepsin and other secretions necessary to dissolve my food—I do not want anybody to chew for me, and I want to do my own digesting; and I can assure you that I derive a great deal of comfort from both these performances. Even if this is not the best, I like to have the pleasure of doing it myself. In this respect I do not believe in socialism; that is one of the things that I do not want the state to do for me unless I become incapacitated. If you want to loose your teeth as soon as possible, eat prechewed foods, and if you want to ruin your stomach, eat predigested foods. The Lord knows that most of us can digest our own starch, and do not need the chemist to put sulfuric acid in it to dissolve it for us. All the efforts of the makers of the more modernized foods seem to rest on the assumption that we have no teeth and no gastric juices, and both of these assumptions are erroneous. True dental hygiene demands normal, natural foods on which the teeth and the stomach can exercise themselves, in order to perform their normal functions; and I think, in saying this, that I am preaching not only good physiology, but plain common sense, and am voicing the experience of everyone who has taken the trouble to look into this matter at all.

My next theme is how to give the teeth proper food for mastication, which is as important as having the mill to masticate with. The miller may have a good upper and lower millstone, but if he tries to grind pebbles into flour, he will have hard work. So, after we bring into good condition the millstones which nature has furnished, let us bring good grits to the mill. That is the reason why I have made the keynote of my fight for public health, good nutritious, unadulterated food, because, without this, health is impossible; and in the same way the dentists of this country should do their share in this great warfare for the welfare of the public. This follows as a natural corollary to that which I have already illustrated. After we have secured the nutritious food which is necessary to the happiness and health of man, we must have proper mastication. I am not so extreme on this question, however, as Mr. Fletcher is. I believe in his doctrine, but, like the rest of us cranks, perhaps he emphasizes it too much. Still, I believe in thorough, slow mastication, in careful selection and a proper and generous quantity of food. I am not an apostle of starvation: I do not believe that humanity will be helped by starving. I am a great admirer of Upton Sinclair, but when he says that fasting will cure every form of disease which attacks humanity, I say, "What are you going to do with the man who is dying of hunger? Thousands are dying from that, and do you propose to preach that doctrine to them?" Fasting is good for a man who habitually overeats, and a great many of us are too often and too well fed.

A few years ago I addressed a company of farmers, worth fifteen millions or more each, and as I looked at them at Sherry's, sitting around the table drinking champagne which cost as much as the milk they produced—when you visit one of these farmers they set before you milk and champagne, and suggest that you help yourself, one costing as much as the other—I felt that they were in danger. I am not speaking disrespectfully of these men, but every one of them was eating himself into an early grave. It is said that Napoleon ate himself into Waterloo; that had he not been such a gourmand and had he not dulled his faculties by overeating, the battle of Waterloo would not have been lost by him. And these men were Napoleons of finance, and were eating themselves into their Waterloo as fast as they could. For that kind of people, fasting is good. The man who really works, who puts forth exertion every day to the utmost, is not the man to preach starvation to, but the man who "sits still," as Mrs. Browning says, "in easy chairs and damns the general world for standing up"—he is the man to recommend starvation to. Physiologists all over the world advise us to eat pure food and masticate it well, and that is the doctrine I preach to you.

### PREVENTION AS THE AIM.

Let me ask for a moment what is to become of the medical and the dental professions in that day, which I hope is not far off, when disease will be banished from this world, and when all will be born, not with actually sound *teeth*, but with sound germs for perfect teeth, which are not found in all newly-born today. We inherit the tendency to caries, as all know. What is to become then of the physician and the dentist? I have said this summer in lectures before Chautauquas that only two really learned professions are necessary to the happiness of mankind, the one, the profession of agriculture, the other the teaching profession, to which we all belong—every good citizen is a teacher. When the propaganda for public health has had its full fruition there will be no disease, and as far as we are informed at the present we would have no need of physicians; and when my propaganda for good teeth has reached its fruition we will have no need for the dentist as he works today; but, as we shall see immediately, physicians and dentists will still be needed in another way. Then, I, hope, when we are all perfectly well and need no doctors, and when we have good teeth and need no dentists, we will not need many preachers. We will be so good that we will not need any body to tell us how to get to heaven—we will just get there naturally; and when we do not need any ministers of the gospel, we will not need any lawyers; we will not break the laws. In short, in the Wiley millennium there will be no physicians, no dentists, no ministers, no lawyers, just farmers and teachers. We will be employed then for the prevention of disease of the body and the mouth, and then, in those future days of ideal socialism, the physician and the dentist will be employed as officials of the state to look after the health of the state, and their salaries will be in proportion to the good teeth and the health of the people, and as people become sick, and lose their teeth, the salaries of the dentist and the physician will diminish! That is the way in which we will employ the physician and the dentist of the future; but you need not be afraid that you will lose your jobs tomorrow. Somebody will have toothache tomorrow in spite of my talk, and that ideal time is still far off. It is not going to come through any battle of Armageddon, but it will come through the preaching of the gospel of health and the teaching of this generation and the next to care for their children's teeth and health, and not have them die by the thousands, as they are dying today in the United States. Nearly a thousand babies not yet one year of age have closed their eyes today in this country of ours, and this can be avoided when the mothers and fathers are educated to understand the principles of hygiene, and know how to bring healthy children into the world, as well as care for them after they are born; these will grow up to be healthy men and women, and gray hairs will be as common then as bald heads are today, and we shall not look with disfavor on age, as we do at the present time. Then we shall have four grandfathers and grandmothers, instead of none, and happiness and prosperity will come to this country; not wealth for the few and poverty for the many, but an even distribution of the wealth of the country, according to the individual's efforts and usefulness, and every one will have an opportunity to make an effort for the good of the world, and health and happiness and contentment will be the lot of humanity.

